# A randomized controlled trial comparing conservative versus surgical treatment in patients with foot drop due to peroneal nerve entrapment: results of an internal feasibility pilot study

**DOI:** 10.1186/s40814-023-01407-x

**Published:** 2023-10-31

**Authors:** Christophe Oosterbos, Sofie Rummens, Kris Bogaerts, Anaïs Van Hoylandt, Sophie Hoornaert, Frank Weyns, Annie Dubuisson, Jeroen Ceuppens, Sophie Schuind, Justus L Groen, Robin Lemmens, Tom Theys

**Affiliations:** 1 Research Group experimental Neurosurgery and Neuroanatomy KULeuven and the Leuven Brain Institute, Leuven, Belgium; 2 Department of Neurosurgery, University Hospitals Leuven, Leuven, Belgium; 3 Department of Physical Medicine and Rehabilitation, University Hospitals Leuven, Leuven, Belgium; 4 Department of Public Health and Critical Care, I-BioStat, KU Leuven, Belgium and I-BioStat, UHasselt, Belgium; 5 Department of Neurosurgery, Ziekenhuis-Oost Limburg, Genk, Belgium; 6 Neurosciences, Faculty of Medicine and Life Sciences, UHasselt, Hasselt, Belgium; 7 Department of Neurosurgery, University Hospitals Liège, Liège, Belgium; 8 Department of Neurosurgery, Algemeen Ziekenhuis Groeninge, Kortrijk, Belgium; 9 Department of Neurosurgery, Erasme Hospital, Brussels, Belgium; 10 Leiden Nerve Center, Department of Neurosurgery, University of Leiden, Leiden, the Netherlands; 11 KU Leuven–University of Leuven, Department of Neurosciences, Experimental Neurology, Leuven, Belgium; 12 VIB, Center for Brain & Disease Research, Laboratory of Neurobiology, Leuven, Belgium; 13 Department of Neurology, University Hospitals Leuven, Leuven, Belgium

**Keywords:** Randomized controlled trial, Foot drop, Peroneal nerve, Neurolysis, Conservative treatment, Feasibility

## Abstract

**Background:**

Based on the lack of literature to support any treatment strategy in patients with foot drop due to peroneal nerve entrapment, a prospective study randomizing patients between surgery and conservative treatment is warranted. Since studies comparing surgery to no surgery are often challenging, we first examined the feasibility of such a randomized controlled trial.

**Methods/design:**

An internal feasibility pilot study was conducted to assess several aspects of process, resource, management, and scientific feasibility. The main objective was the assessment of the recruitment rate. The criterion to embark on a full study was the recruitment of at least 14 patients in 6 participating centers within 6 months. Cross-over rate, blinding measures, training strategies, and trial assessments were evaluated. The trial was entirely funded by the KCE Trials public funding program of the Belgian Health Care Knowledge Centre (ID KCE19-1232).

**Results:**

The initial duration was prolonged due to the COVID-19 pandemic. Between April 2021 and October 2022, we included 19 patients of which 15 were randomized. Fourteen patients were treated as randomized. One drop-out occurred after randomization, prior to surgery. We did not document any cross-over or accidental unblinding. Training strategies were successful. Patients perceived the quality of life questionnaire as the least relevant assessment. Assessment of ankle dorsiflexion range of motion was prone to interobserver variability. All other trial assessments were adequate.

**Discussion:**

Recruitment of the anticipated 14 patients was feasible although slower than expected. The Short-Form Health Survey (SF-36) and assessment of ankle dorsiflexion range of motion will no longer be included in the full-scale FOOTDROP trial.

**Conclusion:**

The FOOTDROP study is feasible.

**Trial registration:**

ClinicalTrials.gov, identifier NCT04695834. Registered 4 January 2021.

**Supplementary Information:**

The online version contains supplementary material available at 10.1186/s40814-023-01407-x.

## Key messages regarding feasibility

- Studies randomizing patients between surgery and non-invasive treatment, allowing only late cross-over, are challenging in terms of patient recruitment.
- Given a multidisciplinary collaboration between all specialism involved, the FOOTDROP trial is feasible. Bias towards surgical or non-invasive treatment in patients with foot drop due to peronel nerve entrapment is real.
- The full study should only be initiated in centers without significant bias towards any treatment strategy and only if a multidisciplinary collaboration is possible.

## Introduction

Peroneal neuropathy is a well-established cause of foot drop [1], often causing gait difficulties and increased risk of falling [2]. In previous research [3], we proposed to classify peroneal neuropathies as idiopathic, idiopathic with established risk factors (e.g., weight loss), and non-idiopathic peroneal neuropathies (e.g., trauma) to account for the wide variety of causes of peroneal neuropathy. We defined peroneal nerve entrapment as idiopathic neuropathies with and without risk factors.

So far, patients with peroneal nerve entrapment have been treated conservatively or through neurolysis of the peroneal nerve. Conservative treatment focuses on gait rehabilitation, proprioceptive training, strengthening of the dorsiflexion muscles of the ankle, passive mobilization of the ankle and foot and stretching of calf muscles (prevention of contractures). A central issue in the management of peroneal nerve entrapment is the lack of evidence to support either treatment strategy [3]. Furthermore, in an international survey among specialists, we documented important variations in daily management of these patients as a consequence of this lack of high-quality evidence [4].

To optimize patient care, a randomized controlled trial comparing both treatment strategies is warranted. Recently, we published the study protocol of our randomized controlled trial [5]: “A prospective, multi-center, randomized, parallel-group controlled trial to compare conservative versus surgical treatment of foot drop in peroneal nerve entrapment” (Acronym FOOTDROP: Follow-up and Outcome of Operative Treatment with Decompressive Release Of the Peroneal nerve). The FOOTDROP study is the first of its kind to compare a non-invasive treatment strategy with neurolysis of the peroneal nerve.

Nevertheless, studies comparing surgery to no or delayed intervention are challenging. Little is known about the epidemiology of these patients [3], making the recruitment potential of a study uncertain. Taking physician bias into account [4], a no cross-over policy to surgery within the first 9 months after randomization may hamper optimal recruitment. In addition, in the absence of high-quality evidence, establishing relevant study outcome measures and endpoints is difficult.

We therefore studied the feasibility of this multicenter randomized controlled trial for patients with foot drop due to peroneal nerve entrapment in an internal feasibility pilot study in 5 centers in Belgium and 1 center in the Netherlands. These centers are Leiden Nerve Center, AZ Groeninge Kortrijk, University Hospitals Leuven, University Hospitals Liege, ZOL Genk and University Hospitals Erasme Brussels.

## Methods/design

### Objectives

Pilot study objectives were categorized as process feasibility, resource feasibility, management feasibility or scientific feasibility as suggested in the literature on pilot study methodology [6–8]. The progression criterion to embark on a full study was to randomize at least 14 patients in the 6 centers overall, over a period of 6 months.

#### Process feasibility

The main pilot study objective was the assessment of recruitment rate. The overall recruitment rate (proportion of patients randomized/proportion of eligible patients) was determined, as well as recruitment rate per clinical center. Since the current literature lacks reliable data to assess a realistic recruitment rate, the optimal local recruitment potential was defined based on physician experience and daily practice.

Reasons for ineligibility and participation refusal were documented. The no cross-over policy was tested during the pilot study (evaluation by percentage of actual cross-over). Furthermore, we wanted to assess whether the study assessments could be completed within a reasonable amount of time and whether the study visits created a significant participant burden. For these purposes, we developed a feasibility study questionnaire that was completed by all participants at 6 weeks and 3 months after randomization (Additional file 1: Appendix 1).

#### Resource feasibility

We aimed to evaluate the clinical center’s motivation and (administrative) capacity to carry through with project-related tasks [6]. We wanted to test whether all centers were able to establish a multidisciplinary study team and whether all administrative requirements for patient enrollment could be timely fulfilled (= within 6 weeks after the site initiation visit). A multidisciplinary study team was defined as a study team consisting of a neurosurgeon, an electrophysiologist, at least one blinded outcome assessor, and a clinical trial assistant. Resource-related issues were discussed during Trial Steering Committees and regular communication with the principal investigators. Compliance with the study protocol and regulations of good clinical practice were controlled during study monitoring visits conducted at each site that randomized at least one patient. We checked whether all pre-screenings and screenings were correctly documented in the appropriate logs. We controlled whether informed consent was obtained, whether data entry occurred correctly, and whether paper source documents were used as intended.

#### Management feasibility

Blinding measures were evaluated by looking at the number of unblinding incidents. Blinded outcome assessors were to report each unblinding event to the principal investigator. Since the pilot study was conducted during the global COVID-19 pandemic, we wanted to evaluate the influence of COVID-19 on patient recruitment by plotting the number of pre-screenings/screenings against the number of patients hospitalized due to COVID-19 at the Sponsor’s site.

#### Scientific feasibility

We wanted to assess whether the chosen assessments were relevant from a patient’s perspective. Patient’s feedback on trial assessments was collected via the feasibility questionnaire (Additional file 1: Appendix 1) that was completed at 6 weeks and 3 months after randomization by trial participants. Finally, the reliability of the different study assessments was tested throughout the pilot study. Issues regarding the study assessments were discussed during the Trial Steering Committees.

### Trial registration and ethical approval

The FOOTDROP study was registered on ClinicalTrials.gov with the identifier NCT04695834 on 4 January 2021 (https://clinicaltrials.gov/ct2/show/NCT04695834). The KCE trials number is KCE19-1232 and the Sponsor study number is S62895. The FOOTDROP study received ethical approval on 30 March 2021 in all 5 participating centers in Belgium and on 06 July 2021 from the Ethics Committee in Leiden, The Netherlands.

### Summary of study design

We refer to the protocol paper for the full details but will summarize the main criteria and assessments [5]. The feasibility study was designed to be an internal pilot study meaning that all study visits, trial assessments, and endpoints were identical to those of the full study. This allows for pilot study data integration into the full study data set. This manuscript closely adheres to the Consort 2010 statement extension to randomized pilot and feasibility trials [9].

Patients fulfilling the eligibility criteria (see Table 1) were included at the screening visit after informed consent was obtained by the treating physician. All patients were treated in a non-invasive manner until the randomization visit, 10 ± 4 weeks after symptom onset. If foot drop persisted at the randomization visit, patients were 1:1 randomized between prolonged non-invasive treatment and neurolysis of the peroneal nerve within 1 week after randomization. No cross-over to surgery was allowed within the first 9 months after randomization. After this point, surgery was allowed in case of persisting foot drop. A no cross-over policy allows for sufficient time for nerve recovery in the nonsurgical treatment group [10]. A prolonged conservative treatment is supported by an established clinical equipoise [3] and important variations in daily practice [11]. This is extensively discussed with patients prior to enrolment.

**Table 1:** Eligibility criteria

Inclusion criteria - EDX-documented peroneal nerve entrapment with persisting (10 ± 4 weeks) foot drop (MRC ≤ 3)
- Age ≥ 18 years
- Imaging (ultrasound/MRI) to exclude a compressive mass at the level of the fibular head
- Written informed consent
*Exclusion criteria*
- Posttraumatic/iatrogenic peroneal nerve injury
- Peroneal neuropathy due to compressive mass
- Peroneal neuropathy at other sites than the fibular head
- Bilateral peroneal nerve entrapment
- Psychiatric illness
- Pregnancy
- Previous foot drop
- Permanently bedridden subjects
- Neurological/musculoskeletal history with impact on assessment and/or gait analysis
- Incapacitated to participate in physiotherapy program (mental/physical illness)
- Planned (e)migration within 1 year after randomization

**Fig. 1:**
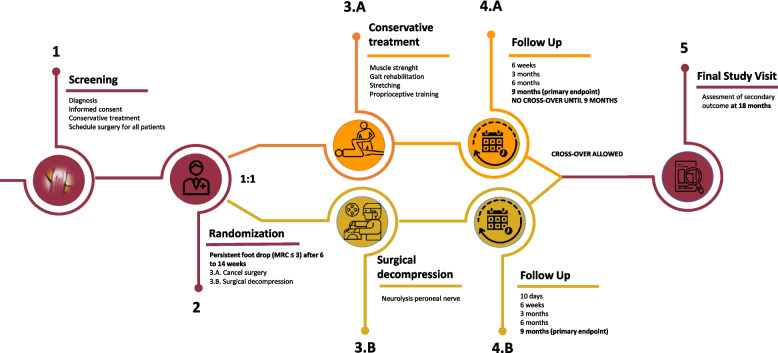
Trial flow [5]

All patients were evaluated at fixed time points after randomization: at 6 weeks, 3 months, 6 months, 9 months, and 18 months. In addition, patients treated surgically were evaluated 10 days after surgery (Fig. 1). Trial assessments were conducted by blinded outcome assessors. Outcome assessors were preferably physiotherapists but could be study nurses or physicians. Outcome assessors were trained using training videos of the different trial assessments to reduce interobserver variability and improve data quality. Training occurred during the site initiation visit and was deemed adequate when outcome assessors had no questions about the assessments. The duration of outcome assessor training was close to 1 h and training videos could be rewatched on the website (http://www.footdroptrial.com). To ensure blinding, the following measures were taken: all patients wore long trousers and applied a bandage at the level of the fibular head, to cover a potential scar. Patients were asked to not discuss their treatment modality with the outcome assessors and were reminded of the blinded measures prior to each study visit.

Trial assessments addressed all important aspects of foot drop due to peroneal nerve entrapment (Table 2). A thorough gait assessment consisted of the 6-min walk test (6MWT) [12], the 10-m walk test (10MWT) [13], the Stanmore questionnaire [14], Functional Ambulation Categories (FAC) [15], assessment of the need for foot-ankle orthosis (FAO), and the ability to walk barefoot. Muscle strength for ankle dorsiflexion, hallux extension, and ankle eversion were assessed with the Medical Research Council (MRC) grading scale [16].

**Table 2:** Data collection and outcome measures [5]

Time since randomization	0	10d	6w	3m	6m	9m	18m
6-min walk test	X		X	X	X	X	X
10-m walk test	X		X	X	X	X	X
Isometric dynamometry	X	X	X	X	X	X	X
Muscle strength (MRC-score)	X	X	X	X	X	X	X
Electrodiagnostics (EDX)				X		X	
Functional ambulation categories	X		X	X	X	X	X
Stanmore questionnaire	X		X	X	X	X	X
Sensory changes	X	X	X	X	X	X	X
QOL questionnaires (EQ5D-5L, SF-36)	X	X	X	X	X	X	X
Return to work			X				
Surgical complications		X	X				X
Treatment record	X		X	X	X	X	X
Ability to walk barefoot	X		X	X	X	X	X
Need for orthosis	X		X	X	X	X	X
WPAI questionnaire	X		X		X		
Ankle dorsiflexion range of motion	X	X	X	X	X	X	X

*d *Days, *w* Weeks, *m *Months

Isometric dynamometry [17] was used to objectively measure ankle dorsiflexion strength. Active and passive ankle dorsiflexion range of motion [18] were measured, using a goniometer, with the patient’s knee flexed and extended. Electrodiagnostics (EDX), including nerve conduction studies and electromyography [19], were repeated in both groups at 3 months and 9 months after randomization. Quality of life (QOL) was assessed with the EQ5D-5L [20, 21] and 36-Item Short Form Health Survey (SF-36) [22]. Surgical complications [23] were registered and a treatment record was made for all patients. Sensory changes in the skin innervated by the peroneal nerve were assessed using a 3-point scale (no recovery, partial recovery, and full recovery) as implemented by Broekx et al. [24]. A health economic assessment consisted of the Work Productivity and Impairment Questionnaire (WPAI) [25] and the registration of return to work 6 weeks after randomization.

The primary endpoint of the FOOTDROP study is the difference in distance covered during the 6MWT (6MWD) between randomization and 9 months after randomization. The primary and (key) secondary endpoints were not analyzed during the feasibility pilot study and are therefore not discussed in this manuscript. For additional information, we refer to the study protocol [5].

Study data were collected and managed using REDCap electronic data capture tools hosted at University Hospitals Leuven [26, 27]. REDCap (Research Electronic Data Capture) is a secure, web-based software platform designed to support data capture for research studies, providing (1) an intuitive interface for validated data capture; (2) audit trails for tracking data manipulation and export procedures; (3) automated export procedures for seamless data downloads to common statistical packages; and (4) procedures for data integration and interoperability with external sources.

### Statistical analysis

Feasibility metrics were assessed using appropriate descriptive statistics. No hypothesis testing was planned in this pilot study. The pilot study is considered as the first phase of the substantive study and pilot study data will be integrated into the full study.

## Results

### Process feasibility

#### Recruitment and recruitment rate

**Fig. 2:**
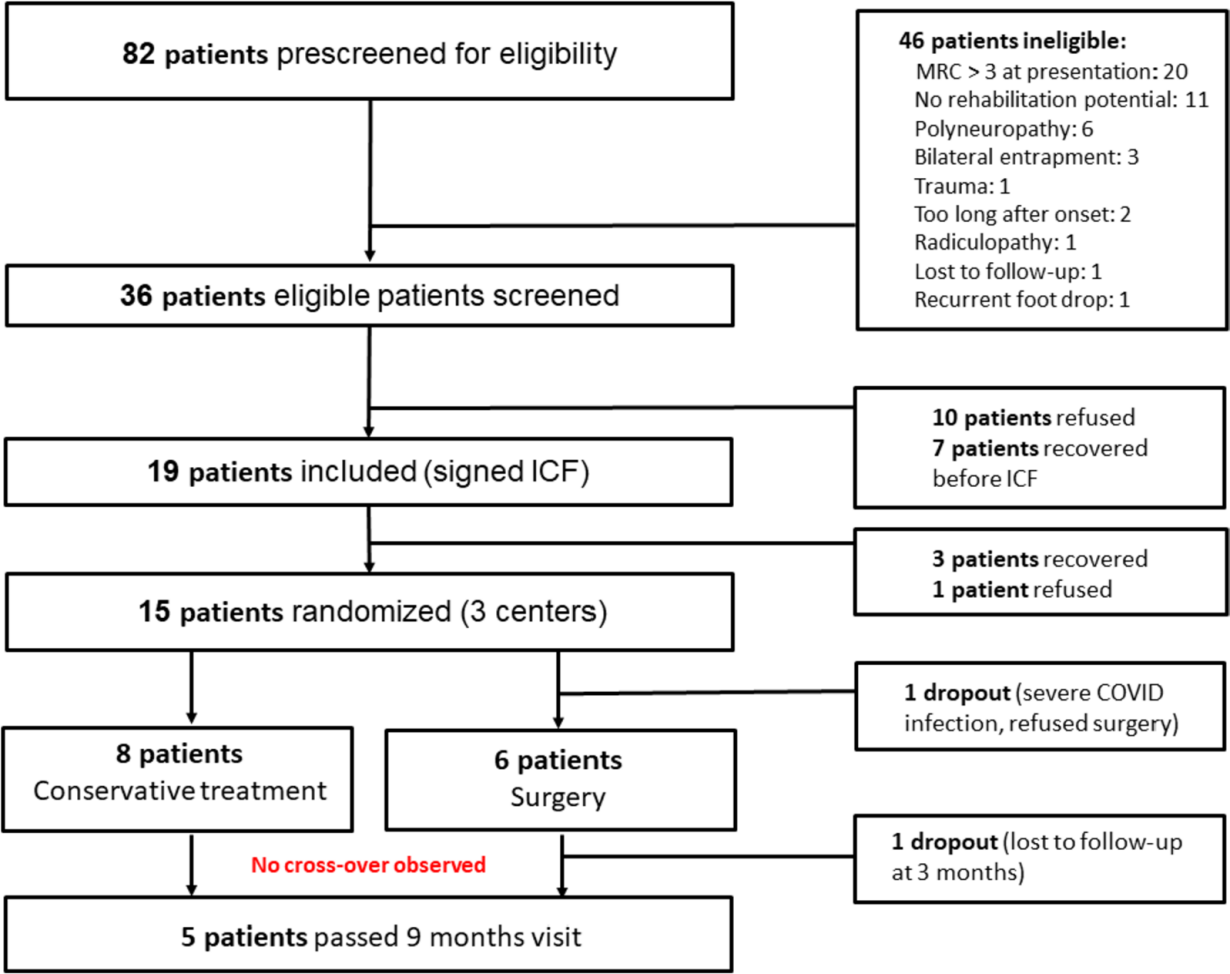
Recruitment throughout the feasibility pilot study

Between April 2021 and October 2022, 82 patients were prescreened for trial participation. The most frequent reasons for ineligibility were: limited loss of ankle dorsiflexion strength (*n* = 20), incapacity to participate in a rehabilitation program (*n* = 11), and presence of polyneuropathy (*n* = 6) (see Fig. 2).

More than half (52.8%) of the 36 patients fulfilling the eligibility criteria were included. Ten eligible patients (27.8%) refused participation in the study. Known reasons for refusal were bias towards surgery (*n* = 2), bias against physiotherapy (*n* = 1), unwillingness to be randomized (*n* = 1), study burden anticipated as too high (*n* = 2) and logistic problems (*n* = 1). Seven patients considered participation but recovered before informed consent was signed.

Fifteen patients were randomized in 3 centers over a period of 18 months, corresponding to an overall recruitment rate of 41.7%. The recruitment rate per center is reported in Table 3.

**Table 3:** Recruitment throughout the pilot study per center

	^a^prescreenings	^a^eligible patients	^a^inclusions	^a^randomizations	Recruitment rate
Center 1	51	13	12	10	76.9 %
Center 2	8	6	3	3	50.0 %
Center 3	4	4	2	2	50.0 %
Center 4	10	7	1	0	0.0 %
Center 5	9	6	1	0	0.0 %
Center 6	0	0	0	0	0.0 %
Overall	82	36	19	15	41.7%

^a^number of

In total, 10 out of 36 (27.8%) eligible patients recovered within 14 weeks of symptom onset (defined as an MRC-score for ankle dorsiflexion ≥ 4). One patient refused randomization and preferred conservative treatment over surgery. Fourteen out of fifteen randomized patients were treated as randomized (93.3%). The fifteenth patient was randomized to surgery but suffered from a severe COVID-19 infection immediately after randomization. The patient refused surgery and dropped-out (Fig. 2; recruitment throughout the pilot feasibility study).

#### Evaluation of the no cross-over policy

In total, 8 patients were randomized to conservative treatment. In this patient group, no cross-over to surgery was observed (0.0% crossover rate).

#### Participant burden

All patients understood the goal of the study and all patients acknowledged that they received enough information about the trial and the trial flow. All patients agreed that the instructions during the assessments were clear and understandable. At 6 weeks after randomization, no patient considered the study visits to be too time-consuming. At 3 months, one patient experienced the study visits as too time consuming. At 6 weeks, median time for completion of the trial assessments was 60 minutes (minimum of 60 min, maximum of 90 min). At 3 months, median time for completion of the trial assessments was 70 min (minimum of 30 min, maximum of 120 min). Only a minority of patients perceived the different trial assessments as irrelevant. Additional file 2: Appendix 2 provides an overview of the results of the pilot study questionnaire.

### Resource feasibility

The study was successfully initiated in all six pilot study centers. All centers complied with administrative requirements to start recruiting patients (=green light); see Table 4.

**Table 4:** Resource feasibility

	Successful initiation?	Interval EC approval–SIV	Interval SIV–green light	Multidisciplinary study team?
Center 1	Yes	7 days	22 days	Yes
Center 2	Yes	9 days	33 days	Yes
Center 3	Yes	21 days	44 days	Yes
Center 4	Yes	22 days	20 days	Yes
Center 5	Yes	21 days	36 days	Yes
Center 6	Yes	122 days	124 days	No CTA

*EC* Ethics committee, *SIV* Site initiating visit, *CTA* Clinical trial assistant

One center was not able to establish a multidisciplinary study team. The same center did not receive ‘green light’ to start screening patients within 6 weeks after the trial assessments. Monitoring visits were conducted in center 1, center 2, and center 3. Inappropriate use of the screening logs in center 2 and center 3 became apparent. Not all ineligible patients or negative screenings were properly logged. No important issues regarding the informed consent, use of the paper source documents, and REDCap database were observed. No major protocol violations occurred.

### Management feasibility

#### Feasibility of blinding measures

Blinding measures were successfully installed with no reported cases of accidental unblinding. At 6 weeks after randomization, 1 patient perceived the blinding measures as bothersome. No one perceived the measures as bothersome at 3 months after randomization (Additional file 2: Appendix 2).

#### Influence of the COVID-19 pandemic

**Fig. 3:**
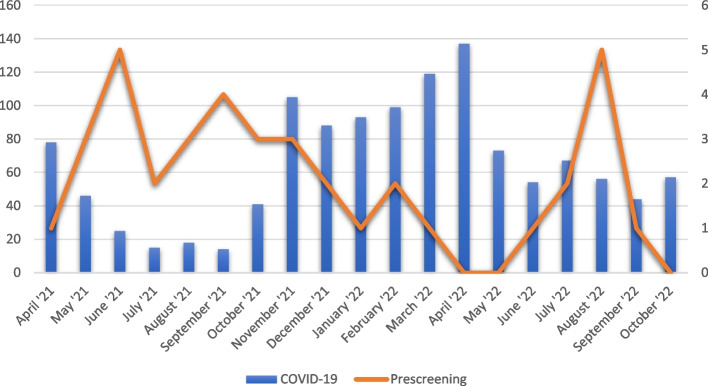
The influence of the COVID-19 pandemic on patient recruitment. The graph shows the number of patients hospitalized due COVID-19 at UZ Leuven (blue bars, scale on left *y*-axis) and the number of (pre)screenings in UZ Leuven (orange line, scale on right *y*-axis)

The duration of the pilot study was prolonged due to the COVID-19 pandemic. Figure 3 plots the number of patients hospitalized in UZ Leuven due to COVID-19, against the number of (pre)screenings in UZ Leuven. A negative correlation is apparent.

### Scientific feasibility

#### Trial assessments from a patient’s perspective

Nine patients completed the trial assessment questionnaire at 6 weeks and at 3 months. A large majority of patients experienced the 6MWT, 10MWT, repeated electrodiagnostics, and isometric dynamometry as relevant to their health problem. This was true both at 6 weeks and 3 months after randomization (Table 5). Opinions regarding the health-related QOL questionnaires and the WPAI questionnaire were more divided. Some patients felt they had to complete too many questionnaires throughout the study (see also Additional file 2: Appendix 2). One patient thought the SF-36 questionnaire to be excessively long without any additional value over the EQ5D-5L questionnaires.

**Table 5:** Patient feedback on trial assessments

	6 weeks (9 patients)			3 months (9 patients)		
	Relevant	Nor relevant, nor irrelevant	Irrelevant	Relevant	Nor relevant, nor irrelevant	Irrelevant
6MWT	88.9 %	11.1 %	0.0 %	88.9 %	0.0 %	11.1 %
10MWT	66.7 %	22.2 %	1.1 %	88.9 %	0.0 %	11.1 %
EDX	88.9 %	11.1 %	0.0 %	100 %	0.0 %	0.0 %
QOL	55.6 %	44.4 %	0.0 %	55.6 %	33.3 %	11.1 %
WPAI	66.7 %	33.3 %	0.0 %	66.7 %	11.1 %	22.2 %
Dynamometry	88.9 %	11.1 %	0.0 %	77.8 %	11.1 %	11.1 %

*6MWT* 6-min walk test, *10MWT* 10-m walk test, *EDX* Electrodiagnostics, *QOL* Quality of life, *WPAI* Work productivity and activity impairment questionnaire

#### Reliability of the trial assessments

Training for all assessments was successful with the exception of training for ankle dorsiflexion range of motion. Despite attempts for more strict instructions, important interobserver variability was observed. Repeated measurements within patients yielded very different values and important variations in reported values between different centers were observed. After retraining and adapting the measurement strategies, issues kept occurring.

## Discussion

We report the feasibility of a prospective, parallel-group, randomized controlled trial for patients with foot drop due to peroneal nerve entrapment.

As 15 patients were randomized, the go criterion to embark on a full study was reached. However, recruitment was slower than expected. Slower recruitment can partly be explained by the interference of the global COVID-19 pandemic. The COVID-19 pandemic could have impacted patient recruitment in multiple ways. It is possible that patients were reluctant to participate in clinical trials out of fear of COVID-19, or did not seek medical attention for a painless foot drop and recovered spontaneously. Less patients were treated in the outpatient clinic and less healthcare resources were available. It is therefore possible that fewer diagnoses were made. In addition, the operating theatre availability was substantially decreased for non-life-threatening conditions or non-urgent surgical procedures. These findings are in accordance with the literature on the impact of COVID-19 on clinical trial screening rates [28], indicating that there is a negative correlation between the severity of the global pandemic and the number of screenings in clinical trials.

Not all centers were able to randomize patients. Most patients were randomized at one center with a strong multidisciplinary collaboration between the different specialties. We believe that this is key to trial success. Since several specialisms are involved in patient care, the active involvement of all these physicians is required to identify possible eligible trial candidates. Dudley et al. [29] came to the same conclusion in the PREVIEW pilot study (comparing the effectiveness of resuturing versus expectant management for dehisced perineal wounds), also requiring multidisciplinary collaboration. Therefore, the full study will focus on centers where such a collaboration exists or can be realized. Since every diagnosis of peroneal nerve entrapment in our study is based on electrodiagnostics, including the local neurophysiologist in the study team is crucial. The only center not able to timely comply with all administrative requirements for patient recruitment (Table 4), was the only center without support from a clinical trial assistant. Since the administrative burden is substantial, the study should only be considered in centers with support from a clinical trial assistant.

During the pilot study, we learned that both patients and physicians can be biased towards a certain treatment strategy. Three patients refused trial participation due to a strong preference towards either conservative or surgical treatment. One patient dropped out after signing the informed consent because of a strong preference towards non-invasive treatment. During discussions with physicians about eligibility and patient recruitment, it became apparent that in some cases, trial participation was not considered due to bias towards a preferred treatment strategy. These findings are not unique to our pilot study [29–31]. In the report on the PRESTO feasibility randomized controlled trial comparing surgery to conservative treatment for stable thoracolumbar fractures, Cook et al. [31] describe that it is difficult for some ‘opinionated’ surgeons to admit to patients that they do not know what the right answer is. Since this can affect patient recruitment, measures were taken to avoid physician bias in the full study. We performed a feasibility exercise in all new centers based on a feasibility questionnaire that was made together with the international CRO (Contract Research Organization) Keyrus Life Science Belgium. Among other topics, this questionnaire discussed treatment preferences towards any treatment strategy. If it became clear that the bias was too great during the feasibility exercise, the site was no longer considered to be a good trial candidate.

The overall recruitment rate was 41.7%. This recruitment rate is definitely acceptable compared to other pilot studies comparing surgical to conservative treatment, in which the recruitment rate ranges from 26 to 70% [29, 31–33]. Nevertheless, recruitment potential may have been overestimated, regardless of the previous arguments for slower recruitment highlighted above. The results of this feasibility study aid in making a more realistic recruitment strategy. Since few patients are expected to be randomized at a single center, more centers will be initiated. In total, at least 10 eligible patients recovered before randomization. Seven of these patients recovered before informed consent was signed. Data can only be collected during the screening visit if the informed consent form has been signed. In daily practice, this is often not the case since patients want to carefully consider participation. Data were lost for these 10 patients. To tackle this issue, we will train and encourage centers to contact patients about the study prior to the study visit by telephone. Information about the trial is given and the patient brochure is sent to the patient. In this way, patients are already informed about the study at the screening visit, which will facilitate the informed consent procedure. As 28% of eligible patients recovered before randomization (planned at 6 weeks after symptom onset), this time point was well-chosen to exclude a large proportion of patients with rapid spontaneous recovery.

Based on the feasibility pilot study results, blinding and training of outcome assessors is feasible. This is important since it will improve the quality of the data collected. Furthermore, a no cross-over policy may be realistic although the sample size is of course rather small to make any strong statements. Not allowing cross-over is important to account for the potential delay in recovery in patients randomized to conservative treatment.

Overall, trial assessments were well received by both physicians and patients. Assessment of health-related quality of life using the SF-36 questionnaire and assessment of ankle dorsiflexion range of motion were the two exceptions. Based on the pilot study results, the SF-36 questionnaire will no longer be included in the full study. For this particular health problem, there is little added value over the EQ5D-5L questionnaire. Based on patient feedback, quality of life was one of the least relevant trial outcome measures and too many questionnaires had to be completed. Determining (passive) ankle dorsiflexion range of motion would allow for objectifying muscle contractures in patients with long-standing foot drop due to peroneal nerve entrapment. To our knowledge, current literature does not address this potential issue in this patient group. We were, however, unable to measure passive and active ankle dorsiflexion range of motion with good reliability. Repeated measurements in patients yielded very different values and important differences in reported values between centers were observed. This important variability would interfere with data interpretation. Since the added value of determining the range of motion is potentially limited for our research purposes, it was decided during the Trial Steering Committee that this assessment will no longer be part of the full study. Finally, we can state that the 6MWT (the primary endpoint of the study) is feasible for evaluation of patients with foot drop due to peroneal nerve entrapment. Thus far, the test has never been used to evaluate this patient population. Historically, most studies report ankle dorsiflexion muscle strength as the most important outcome measure [3]. However, ankle dorsiflexion strength does not necessarily reflect gait difficulties in this patient population. The 6MWT assesses gait difficulties and takes fatigue into account. The test can easily be standardized, requires no expensive equipment, and can easily be explained to patients. The test was also well received by patients in the pilot study.

## Conclusion

The protocol for a randomized controlled trial for patients with foot drop due to peroneal nerve entrapment comparing surgery with conservative treatment is feasible as shown in these pilot data. Lessons learned during the pilot study will be implemented in the full-scale trial. Last, the results of this feasibility study are relevant for future research and study design in this patient population.

### Supplementary Information

**Additional file 1: Appendix 1.** Feasibility study questionnaire.

**Additional file 2: Appendix 2.** An overview of the results of the pilot study questionnaire.

**Additional file 3: Appendix 3.** Informed consent in English.

## Data Availability

The study results will be owned by the party who generates them. The Sponsor (University Hospitals Leuven) owns the study data. At the end of the study, KCE will receive sponsor-specific study data. This will only occur when data is needed for a full health economic evaluation. This will only be anonymous study data or, where requested by KCE, coded personal data are made available to KCE. The study data shall not be provided to a third party without the prior written approval of KCE, which approval KCE shall not unreasonably withhold or delay and which KCE may subject to specific conditions in order to ensure that the provision of said study data does not have a negative impact on the further performance of the study, the rights granted to KCE under the research agreement and/or the benefit of the Study for the patients and/or the public payers. The contract between the funder (KCE) and sponsor (University Hospitals Leuven) is available online (https://kce.fgov.be/en/kce-trials/calls/2022-kce-trials-2022-investigator-led-call).

## Abbreviations

FOOTDROP: Follow-up and Outcome of Operative Treatment with Decompressive Release Of The Peroneal nerve
KCE: Belgian Health Care Knowledge Centre
MRC: Medical Research Council
EQ5D-5L: 5 Level EUROQoL – 5 Dimensions
SF-36: 36-Item Short Form Health Survey
6MWT: Six-minute walk test
6MWD: Six-minute walking distance
10MWT: Ten-meter walk test
WPAI: Work Productivity and Activity Impairment Questionnaire
EDX: Electrodiagnostics
FAC: Functional Ambulation Categories
QOL: Quality of life
KCE: Belgian Health Care Knowledge Centre
SIV: Site initiating visit
CTA: Clinical Trial Assistant
CRO: Contract Research Organization

## References

[1] Carolus A, Mesbah D, Brenke C. Focusing on foot drop: results from a patient survey and clinical examination. Foot (Edinb).101693. 10.1016/j.foot.2020.101693 33036837

[2] Poppler LH, Yu J, Mackinnon SE (2020). Subclinical peroneal neuropathy affects ambulatory, community-dwelling adults and is associated with falling. Plastic Reconstruct Surg.

[3] Oosterbos C, Decramer T, Rummens S, Weyns F, Dubuisson A, Ceuppens J (2022). Evidence in peroneal nerve entrapment: a scoping review. Eur J Neurol.

[4] Oosterbos C, Rasulic L, Rummens S, Kiekens C, van Loon J, Lemmens R, et al. Controversies in treatment strategies in patients with foot drop due to peroneal nerve entrapment: Results of a survey among specialists. Brain Spine. 2022;2. 10.1016/j.bas.2022.100887 PMC9560709 36248140

[5] Oosterbos C, Rummens S, Bogaerts K, Hoornaert S, Weyns F, Dubuisson A (2022). Conservative versus surgical treatment of foot drop in peroneal nerve entrapment: rationale and design of a prospective, multi-centre, randomized parallel-group controlled trial. Trials.

[6] Tickle-Degnen L (2013). Nuts and bolts of conducting feasibility studies. Am J Occup Ther.

[7] Arain M, Campbell MJ, Cooper CL, Lancaster GA (2010). What is a pilot or feasibility study? A review of current practice and editorial policy. BMC Med Res Methodol.

[8] Thabane L, Ma J, Chu R, Cheng J, Ismaila A, Rios LP (2010). A tutorial on pilot studies: the what, why and how. BMC Med Res Methodol.

[9] Eldridge SM, Chan CL, Campbell MJ, Bond CM, Hopewell S, Thabane L (2016). CONSORT 2010 statement: extension to randomised pilot and feasibility trials. Pilot Feasibility Stud.

[10] Menorca RM, Fussell TS, Elfar JC (2013). Nerve physiology: mechanisms of injury and recovery. Hand Clin.

[11] Oosterbos C, Rasulic L, Rummens S, Kiekens C, van Loon J, Lemmens R (2022). Controversies in treatment strategies in patients with foot drop due to peroneal nerve entrapment: Results of a survey among specialists. Brain Spine.

[12] Agarwala P, Salzman SH. Six-minute walk test: clinical role, technique, coding, and reimbursement. Chest. 2019. 10.1016/j.chest.2019.10.014 PMC7609960 31689414

[13] Perry J, Garrett M, Gronley JK, Mulroy SJ (1995). Classification of walking handicap in the stroke population. Stroke.

[14] Yeap JS, Singh D, Birch R (2001). A method for evaluating the results of tendon transfers for foot drop. Clin Orthop Relat Res.

[15] Holden MK, Gill KM, Magliozzi MR, Nathan J, Piehl-Baker L (1984). Clinical gait assessment in the neurologically impaired reliability and meaningfulness. Phys Ther.

[16] Compston A. Aids to the investigation of peripheral nerve injuries. Medical Research Council: Nerve Injuries Research Committee. His Majesty's Stationery Office: 1942; pp 48 (iii) and 74 figures and 7 diagrams; with aids to the examination of the peripheral nervous system. By Michael O'Brien for the Guarantors of Brain. Brain. 1942;133(10):2838-44. Saunders Elsevier: 2010; pp. [8] 64 and 94 Figures. 10.1093/brain/awq270 20928945

[17] Won YH, Kim KW, Choi JT, Ko MH, Park SH, Seo JH (2016). Correlation between muscle electrophysiology and strength after fibular nerve injury. Neurol Sci.

[18] Fong CM, Blackburn JT, Norcross MF, McGrath M, Padua DA (2011). Ankle-dorsiflexion range of motion and landing biomechanics. J Athl Train.

[19] Marciniak C, Armon C, Wilson J, Miller R (2005). Practice parameter: utility of electrodiagnostic techniques in evaluating patients with suspected peroneal neuropathy: an evidence-based review. Muscle Nerve.

[20] Bouckaert N, Cleemput I, Devriese S, Gerkens S (2022). An EQ-5D-5L Value Set for Belgium. Pharmacoecon Open.

[21] M MV, K MV, S MAAE, de Wit GA, Prenger R, E AS. Dutch Tariff for the Five-Level Version of EQ-5D. Value Health. 2016;19(4):343-52. 10.1016/j.jval.2016.01.003 27325326

[22] McHorney CA, Ware JE, Lu JF, Sherbourne CD (1994). The MOS 36-item Short-Form Health Survey (SF-36): III. Tests of data quality, scaling assumptions, and reliability across diverse patient groups. Med Care.

[23] Ducic I, Felder JM (2012). Minimally invasive peripheral nerve surgery: peroneal nerve neurolysis. Microsurgery.

[24] Broekx S, Weyns F. External neurolysis as a treatment for foot drop secondary to weight loss: a retrospective analysis of 200 cases. Acta Neurochirurgica. 160(9):1847-56. 10.1007/s00701-018-3614-9 29961126

[25] Reilly MC, Zbrozek AS, Dukes EM (1993). The validity and reproducibility of a work productivity and activity impairment instrument. Pharmacoeconomics.

[26] Harris PA, Taylor R, Thielke R, Payne J, Gonzalez N, Conde JG (2009). Research electronic data capture (REDCap)–a metadata-driven methodology and workflow process for providing translational research informatics support. J Biomed Inform.

[27] Harris PA, Taylor R, Minor BL, Elliott V, Fernandez M, O'Neal L (2019). The REDCap consortium: Building an international community of software platform partners. J Biomed Inform.

[28] McDonald K, Seltzer E, Lu M, Gaisenband SD, Fletcher C, McLeroth P (2023). Quantifying the impact of the COVID-19 pandemic on clinical trial screening rates over time in 37 countries. Trials.

[29] Dudley L, Kettle C, Thomas PW, Ismail KM (2017). Perineal resuturing versus expectant management following vaginal delivery complicated by a dehisced wound (PREVIEW): a pilot and feasibility randomised controlled trial. BMJ Open.

[30] Preston NJ, Farquhar MC, Walshe CE, Stevinson C, Ewing G, Calman LA, et al. Strategies to increase participant recruitment to research studies by healthcare professionals. Cochrane Database Syst Rev. 2012(9). 10.1002/14651858.MR000036.pub2 PMC8190980 35658160

[31] Cook E, Scantlebury A, Booth A, Turner E, Ranganathan A, Khan A (2021). Surgery versus conservative management of stable thoracolumbar fracture: the PRESTO feasibility RCT. Health Technol Assess (Winchester, England).

[32] Griffin D, Wall P, Realpe A, Adams A, Parsons N, Hobson R (2016). UK FASHIoN: feasibility study of a randomised controlled trial of arthroscopic surgery for hip impingement compared with best conservative care. Health Technol Assess (Winchester, England).

[33] Hall NJ, Sherratt FC, Eaton S, Reading I, Walker E, Chorozoglou M (2021). Conservative treatment for uncomplicated appendicitis in children: the CONTRACT feasibility study, including feasibility RCT. Health Technol Assess (Winchester, England).

