# Supplementary material for: A randomized controlled trial comparing conservative versus surgical treatment in patients with foot drop due to peroneal nerve entrapment: results of an internal feasibility pilot study
Source: Pilot Feasibility Stud. 2023 Oct 31;9:181. doi: 10.1186/s40814-023-01407-x (PMC10617035; doi:10.1186/s40814-023-01407-x)
Supplement: Supplementary file 1 — Additional file 1: Appendix 1. Feasibility study questionnaire. [file 40814_2023_1407_MOESM1_ESM.pdf]

Dear patient,

The foot drop research team thanks you for your participation in the trial! During the pilot phase, we would like you to complete following questionnaire regarding the study design and trial assessment after the 6 weeks and 3 months study visit.

We value you opinion and we will use your feedback to adapt the trial as good as possible to the needs of the patients. Completing the questionnaire will only take a few minutes of your time.

We thank you again for completing this short survey!

The FOOT DROP research team

GENERAL QUESTIONS

	YES	NO
**I understand the goal of the study.**	☐	☐
**The instructions during the assessments were clear and understandable.**	☐	☐
**I received enough information about the trial and the trial flow.**	☐	☐

BLINDING PROCEDURE

During the study visit, you were asked to not discuss treatment with your assessor. Furthermore, you were asked to were long trousers and to wear a bandage at the level of the need, so that the investigator cannot deduct if you had surgery based on the postoperative scar. These measures were installed to guarantee an objective evaluation by the outcome assessor. **Did you receive these measures as bothersome?**

- ☐ **Not bothersome**
- ☐ **A bit bothersome**
- ☐ **Bothersome**

TRIAL ASSESSMENTS

Can you please indicate if you consider the trial assessments useful in assessing foot drop and relevant in your recovery.

	IRRELEVANT	NOR RELEVANT, NOR IRRELEVANT	RELEVANT
**Assessment of motor function (dynamometer)**	☐	☐	☐
**6-minute walk test**	☐	☐	☐
**10-meter walk test**	☐	☐	☐
**Quality of life questionnaires**	☐	☐	☐
**Professional disability questionnaire**	☐	☐	☐
**Electrodiagnostics (EMG)**	☐	☐	☐

How long did it take to complete all trial assessments? | **………... minutes**

Did the study visit take too much of your time?

- ☐ **Yes**
- ☐ **No**

The six-minute walk test (6MWT) is the most important trial assessment. The investigators asked you to perform the six-minute walk test twice, at the start and end of every study visit. **How did you experience the six-minute walk test?**

- ☐ **I experienced the test as useful and had no problems with completing the 6MWT.**
- ☐ **I experienced the test as useful and had some problems with completing the 6MWT.**
- ☐ **I experienced the test as not useful but had no problems with completing the 6MWT.**
- ☐ **I experienced the test as not useful and had some problems with completing the 6MWT.**

WE VALUE YOUR OPINION!

Please, feel free to share any other remarks regarding your participation in the foot drop trial!
