# Supplementary material for: A randomized controlled trial comparing conservative versus surgical treatment in patients with foot drop due to peroneal nerve entrapment: results of an internal feasibility pilot study
Source: Pilot Feasibility Stud. 2023 Oct 31;9:181. doi: 10.1186/s40814-023-01407-x (PMC10617035; doi:10.1186/s40814-023-01407-x)
Supplement: Supplementary file 2 — Additional file 2: Appendix 2. An overview of the results of the pilot study questionnaire. [file 40814_2023_1407_MOESM2_ESM.pdf]

Evaluation of the different study assessments by participants

Study assessments were evaluated by patients through a pilot study questionnaire, obtained at 6 weeks and 3 months after randomization. Opinions about different study assessments that are not considered standard of care are discussed below. Nine patients answered the pilot study questionnaire at 6 weeks and 3 months. Analysis is based on the data available from the start of the pilot study until the 7th of September 2022 (total of 18 questionnaires).

The six-minute walk test (6MWT)

Overall, the 6MWT was considered relevant, useful and completed without difficulties. At three months after randomization, two patients considered the 6MWT not useful (figure 4). From a patient perspective, the 6MWT seems to be a relevant and well chosen primary endpoint.

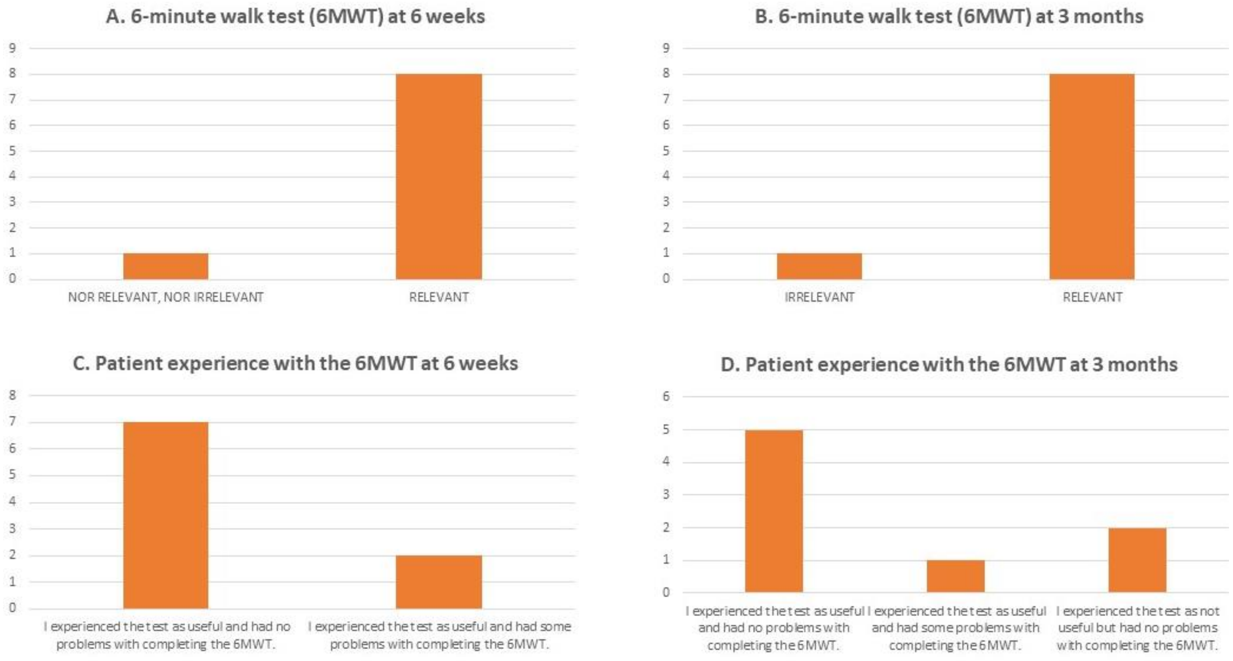

Figure 4 – The six-minute walk test

The 10 meter walk test (10MWT).

The 10-meter walk test was considered relevant by most patients. At 3 months, progressively more patients considered the 10MWT to be relevant compared to 6 weeks (figure 5).

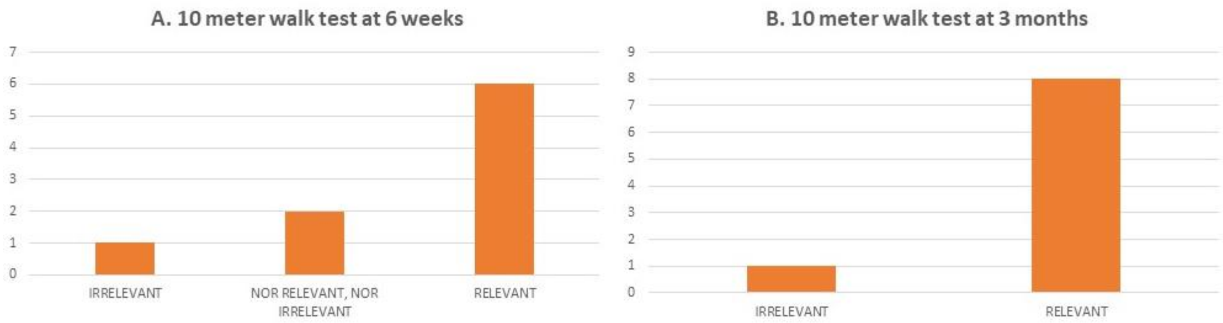

Figure 5 – The 10 meter walk test.

Repeated electrodiagnostics

No patients considered EDX to be irrelevant. EDX is experienced as relevant by all patients at 3 months after randomization. (figure 6). We can conclude that patients find electrodiagnostics important in the follow-up of foot drop.

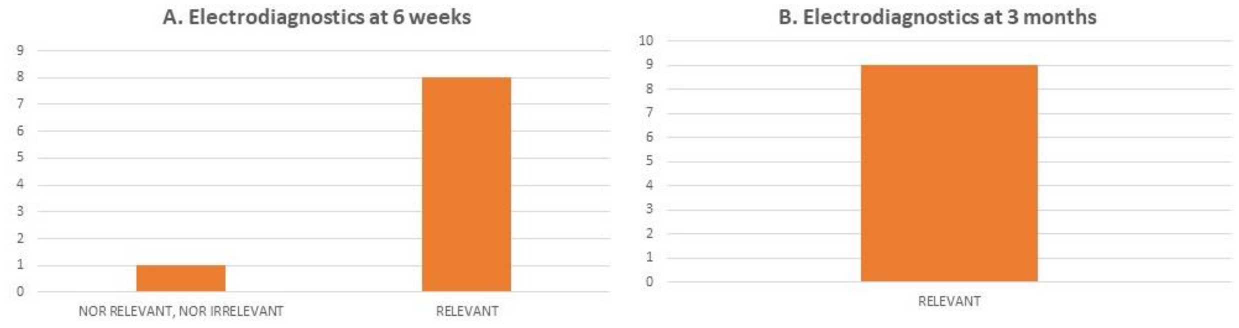

Figure 6 – Electrodiagnostics

Isometric dynamometry

An absolute majority of participants considered the assessment of motor function through isometric dynamometry to be relevant (see figure 7).

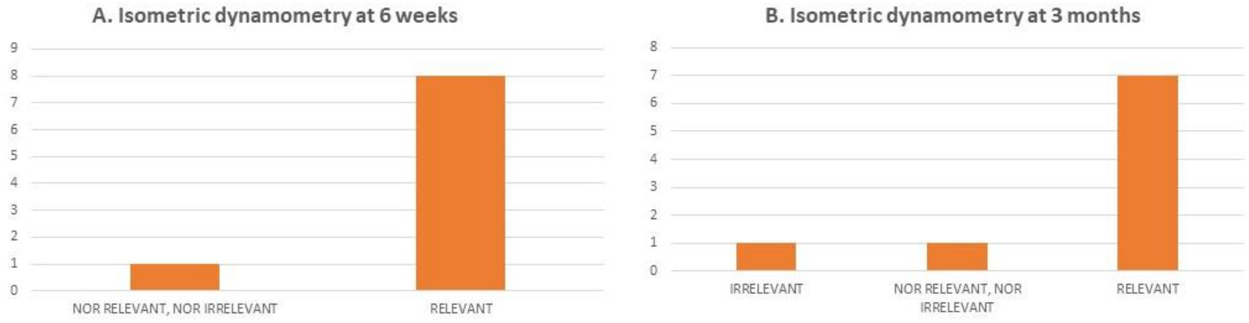

Figure 7 – Isometric dynamometry

Quality of life questionnaires (QoL)

Opinions regarding the quality of life questionnaires were more divided (figure 8). A little more than half of the participants considered the QoL questionnaires to be relevant at both evaluation moments. However, compared to the trial assessments discussed above, more patients considered QoL questionnaires to be less useful at both 6 weeks and 3 months. One patient in Leuven thought the SF-36 to be excessively long without any additional value over the EQ5D questionnaire.

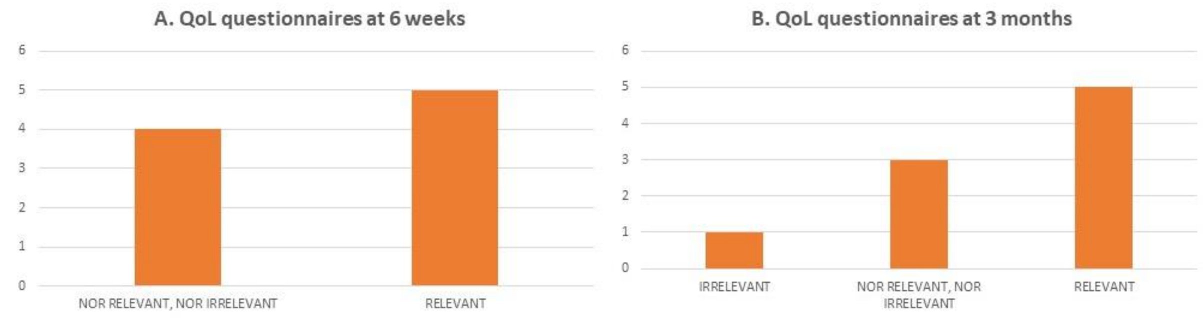

Figure 8 – Quality of life questionnaires

Work productivity and activity impairment questionnaire (WPAI)

Comparable to the quality of life questionnaires, opinions regarding the WPAI questionnaire were divided. However, the largest group of participants still considered the questionnaire to be relevant at both evaluation moments (figure 9).

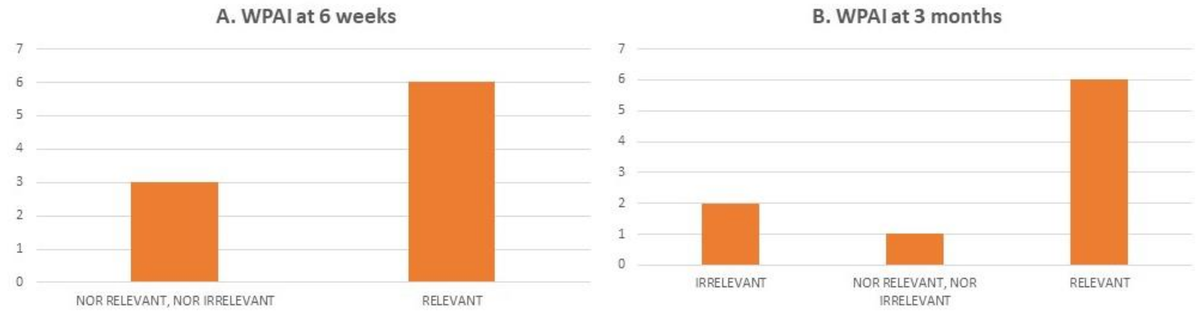

Figure 9 – Professional disability questionnaire

Blinding measures

In the pilot study questionnaire, patients were asked about their experience with the blinding measures (figure 10). Based on participants responses, blinding measures were not experienced as bothersome.

Since this blinding measures are important to guarantee an objective evaluation of patients and no problems are encountered throughout the pilot, blinding measures should be continued throughout the full-scale study.

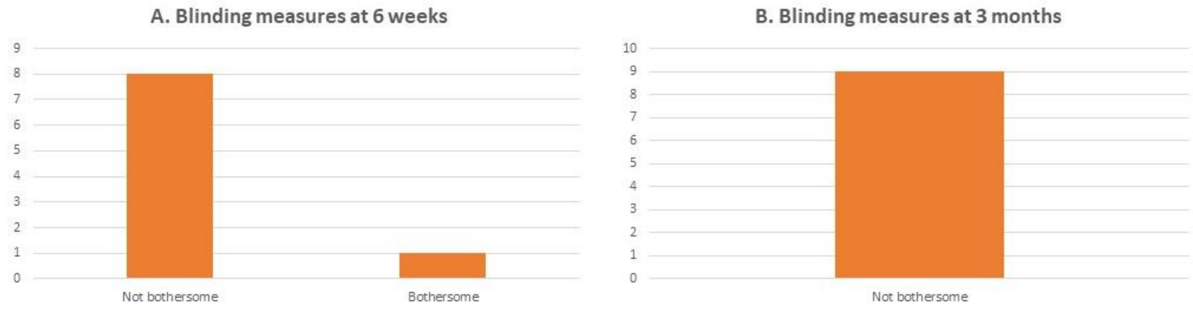

Figure 10 – Blinding measures
