# Supplementary material for: A randomized controlled trial comparing conservative versus surgical treatment in patients with foot drop due to peroneal nerve entrapment: results of an internal feasibility pilot study
Source: Pilot Feasibility Stud. 2023 Oct 31;9:181. doi: 10.1186/s40814-023-01407-x (PMC10617035; doi:10.1186/s40814-023-01407-x)
Supplement: Supplementary file 3 — Additional file 3: Appendix 3. Informed consent in English. [file 40814_2023_1407_MOESM3_ESM.pdf]

Title of the study: **A prospective, multicenter, randomized, parallel-group controlled trial to compare conservative versus surgical treatment in peroneal nerve entrapment.**

A comparative study for surgical and conservative treatment in foot drop due to peroneal nerve entrapment (FOOTDROP trial).

Sponsor of the study:

University Hospitals Leuven
Herestraat 49
3000 Leuven
Belgium

Medical Ethics Committee (EC):

Central EC: Ethical Committee Research UZ Leuven

Local EC's: LOCAL ETHICAL COMMITTEE

Local investigators:

Chief Investigator: Prof. dr. Tom Theys, UZ Leuven

Local principal investigators: LOCAL PRINCIPAL INVESTIGATOR

Clinical trial assistant (CTA): LOCAL CLINICAL TRIAL ASSISTANT

Telephone: TELEPHONE NUMBER LOCAL CLINICAL TRIAL ASSISTANT

I Information vital to your decision to take part

Introduction

You are being invited to take part in a clinical study to evaluate the two most common treatment strategies in patients with foot drop due to peroneal nerve entrapment. Both treatments are considered standard of care and are therefore not experimental.

The sponsor and investigator hope that this study will determine the most effective treatment strategy in this particular disease. There is, however, no guarantee that you will benefit from taking part in this study. Since both treatment strategies are frequently used in daily practice, you are not exposed to additional risks, compared to patients outside of the foot drop trial.

Before you agree to take part in this study, we invite you to take note of its implications in terms of organisation, possible risks and benefits, to allow you to make a decision with full awareness of the implications. This is known as giving "informed consent".

Please read these few pages of information carefully and ask any questions you want to the investigator or his/her representative. There are 3 parts to this document: the information essential to your decision, your written consent and supplementary information (appendices) detailing certain aspects of the basic information.

If you take part in this clinical study, you should be aware that:

- This clinical study is being conducted after having been reviewed by more ethics committees.
- Your participation is voluntary and must remain free from any coercion. It requires the signature of a document expressing your consent. Even after having signed this document, you can stop taking part by informing the investigator. Your decision not to take part or to stop taking part in the study will have no impact on the quality of your care or on your relationship with the investigator.
- The data collected on this occasion are confidential and your anonymity is guaranteed during publication of the results.
- Insurance has been taken out in case you should suffer any damage in connection with your participation in this clinical study.
- You will not incur any charges for the visits/consultations, examinations or treatments specific to this study.
- You may contact the investigator or a member of his/her team at any time should you need any additional information.

Further information about your "Rights as a participant in a clinical study" can be found in appendix III (pages 9-12).

Objectives and description of the study protocol

Recently, you have been diagnosed with a foot drop due to peroneal nerve entrapment. The peroneal nerve provides sensation to the outer part of the lower leg and foot and innervates flexor muscles of the ankle. We are inviting you to take part in a clinical study evaluating treatment strategies in patients with foot drop due to peroneal nerve entrapment, that is to include around 182 patients, both in Belgium and the Netherlands

Current daily management of patients with foot drop due to peroneal nerve entrapment can differ between physicians and centers. Some physicians prefer a conservative treatment focusing on physiotherapy whereas other physicians prefer a surgical decompression. We are inviting you to take part in this study, to investigate which of the two treatment strategies is the most effective. Currently, no good clinical studies comparing both treatment strategies are available.

The outcome of this trial can help the investigators to council and treat future patients in an evidence based manner. If the study shows that one treatment works better than the other treatment, then the study data will also be used to look at whether the new treatment provides better value for money.

All adult patients with confirmed foot drop due to EMG-documented peroneal nerve entrapment without improvement after 6 to 14 weeks are eligible.

This study is a prospective, randomized, parallel-group controlled trial. This means that patients are randomly allocated to one of two treatment groups (surgery or conservative treatment). The outcome of both groups of patients will be compared to examine which treatment strategy is the most effective. Comparison of the two treatment groups will mostly be based on the difference in distance walked during the six-minute walk test between start of the study and nine months later (primary endpoint). During the six-minute walk test, the investigator will instruct you to cover as much distance as possible within 6 minutes.

Course of the study

Your participation in the study will last around 18 months and involve 3 to 4 visits in addition to those involved in your care if you do not take part in the study.

Similarly, several additional examinations or procedures will be performed related to the study.

Since your participation in the study is part of the care of your clinical situation, some of the visits and examinations we will describe are part of the normal care provided in your hospital, while others are offered by the study.

Screening phase:

Since every foot drop has the intrinsic capacity to recover, all included patients will be treated conservatively immediately after diagnosis.

Randomization (start of the trial):

If foot drop does not recover within 6 to 14 weeks after onset of symptoms, you will be randomly allocated to either maximal conservative treatment or surgical decompression of the peroneal nerve. This is called randomisation. If applicable, surgery will be planned within one week after randomization. Both treatment options are equally likely. At the moment of randomization, baseline assessments will be conducted.

Study phase:

After randomization, follow-up is scheduled at fixed timepoints: 6 weeks, 3 months, 6 months, 9 months and 18 months after randomization. The most important study visit is organised 9 months after randomization (primary endpoint). If you had surgery, a standard of care postoperative control will be planned 10 days after surgery. Assessments are mostly limited to standard of care during the postoperative visit. In daily practice, it is considered standard of care to evaluate a patient 1 to 2 times after diagnosis (depending on the clinical evolution and treating physician). The other 3 to 4 visits are therefore considered study visits. During the study visits, your examiner will not know to which treatment you were randomized. This measure is taken so that the personal beliefs of the examiner can not influence your examination. This process is called blinding.

Cross-over

Only after the study visit 9 months after randomization, patients allocated to conservative treatment can be operated. This is not possible within the study before this point in time. This also means that all patients have the possibly for surgery after 9 months, if deemed necessary.

A schematic overview of the study flow is added to the supplementary information (1. Supplementary information on the organization of the study).

If you agree to take part in the study and meet all the conditions required to be enrolled in the study, you will undergo the tests and examinations described below:

**Assessment**	**What and when?**<br>* not considered standard of care
Clinical evaluation	- Assessment of surgical complications (if applicable) after 10 days, 6 weeks and 18 months<br>- Assessment of muscle strength at the start of the study and after 10 days (surgery), 6 weeks, 3 months, 6 months, 9 months and 18 months<br>- Assessment of sensory changes at the start of the study and after 10 days (surgery), 6 weeks, 3 months, 6 months, 9 months and 18 months<br>-
Gait assessment	6-minute walk test at the start of the study* and after 6 weeks*, 3 months*, 6 months*, 9 months* and 18 months*<br><br>10-meter walk test at the start of the study* and after 6 weeks*, 3 months*, 6 months*, 9 months* and 18 months*

Quality of life	Completion of one questionnaires (EQ5D-5L) at the start of the study*, and after 10 days* (surgery), 6 weeks*, 3 months*, 6 months*, 9 months* and 18 months*
Health economics	Completion of a questionnaire (work productivity and activity impairment questionnaire) at the start of the study* and after 6 weeks* and 6 months*<br>Evaluation of return to work at six weeks*
Electrodiagostics	EMG at 3 months* and 9 months*

The electrodiagnostic investigation evaluates the nerve conduction. Nerve conduction is very similar to an electric signal. Your physician can register and interpret these electric signals with small needles inserted in the muscles and brief electrical pulses. In case of entrapment of the nerve at the knee, the physician will only register a week electric signal (or no signal at all).

Every study visit takes around 50 minutes. An additional 20 minutes are foreseen for EMG at 3 months and 9 months. The trial was designed by experts. Feedback of patients who were treated in the past was taken into account.

Blinding:

The researcher is blinded for your treatment, meaning that he or she does not know if you are operated or not. This is important to make sure the measures as objective as possible. This also means that you cannot discuss your treatment with your investigator and you are requested to wear long trousers and apply a bandage at the knee, regardless of your treatment. The bandages will be available, there is no need to buy your own bandages.

Risks and discomforts

A: Risks associated with the evaluation procedures specific to the study

Both treatment strategies are considered standard of care and are not experimental. Therefore, you are not exposed to more risks, than patients treated outside the study. The surgical procedure is considered safe, and adverse events are rather limited. In up to 5% of patients wound problems, wound infections and postoperative bleedings can be expected. There is a very small chance of damage to the nerve.

There are no additional risks associated with the specific examinations that will be performed in the setting of the study. You can experience some painful needle pricks and electrical sensations during the EMG, similar to the investigation at diagnosis. However, follow-up EMG investigations are limited in number so that the additional burden should be restricted to a minimum.

Notification of new information

It may be that during the course of a clinical study, important new information on the treatment being investigated becomes available. You will be informed of any new element that might affect your decision to continue taking part in this study.

In this case, you will be asked to sign either an addendum to the consent form or a new informed consent form. If, in the light of the new information, you decide to stop taking part in the study, your investigator will see to it that you continue to receive the best possible treatment.

Benefits

You will not personally derive any benefit from taking part in this study, but the results obtained could be important in counselling and treating future patients. Through participation in the trial, optimal follow-up is guaranteed.

The sponsor has arranged to offer you a voucher on completion of the study visits. The voucher has a value of €25 per study visit and will be given twice. One part will be available 9 months after

randomization, and the other part 18 months after randomization. Contact the investigating team for the practical arrangements.

Data management

During the trial, data about you is being collected. These data will be used for research purposes and in connection with scientific and medical publications. We assure you that these data will be handled in a strictly confidential manner. To verify the quality of the study, it is possible that your medical records will be examined by persons subject to professional secrecy and strict conditions.
"If the study shows that one treatment works as well or better than another, the government body funding the study, KCE, may ask for a coded copy of some data from the study to assess which treatment, for example, should be reimbursed or recommended and whether its cost is justified. Your identity will never be known to anyone at KCE.
This data processing is necessary in the public interest to improve public health policy. KCE is responsible for any data processed in the context of this additional study and must comply with data protection rules.
The attached document explains this data processing in detail." (Attachment 2)

Alternative treatment: What happens if

This study compares both available treatment options in peroneal nerve entrapment. There are no alternative treatments that are considered standard of care.

Withdrawal from the study

Your participation is voluntary and you are entitled to withdraw from the study for any reason, without having to justify your decision. Nevertheless, it may be useful for the investigator and for the sponsor of the study to know if you are withdrawing because the constraints of the treatment are too great.

It is possible that your treating physician withdraws you from the trial out of safety concerns or because of insufficient compliance to the study protocol.

Finally, the competent national or international authorities, the ethics committee that initially approved the study or the sponsor may break off the study because the information gathered shows that the one of the treatments is not effective (does not deliver a sufficient level of improvement in the health of the participants), the treatment causes more side effects or more serious side effects than anticipated, or for any other reason.

Treatment after stopping the study

In all these situations of withdrawing from the study, but also when the scheduled participation period has ended, your investigator will assess your state of health and prescribe the best treatment available.

If you consider or wish a operative treatment after stopping the trial, please consult your physician. If there is an indication for operative decompression, this treatment can be installed.

If you take part in this clinical study, we ask you:

- To cooperate fully in the smooth running of this study.
- Not to conceal any information relating to your state of health, the medication you are taking or the symptoms you are experiencing.

You should also be aware that:

To be able to take part in this clinical study and for your safety, you must agree that the investigator informs the various doctors in charge of your health of your participation in this clinical study. You will be required to confirm your agreement in this regard in the consent form.

Contact

If you need further information, but also if you have problems or concerns, you can contact the investigator (NAME LOCAL INVESTIGATOR) or a member of his/her research team (NAME CTA) on the following telephone number (LOCAL TELEPHONE NUMBER).

Outside consulting hours, contact the A&E department of your hospital, indicating that you are taking part in a clinical study. Your records will contain information of use to the on-call doctor in relation to this clinical study.

If you have any questions relating to your rights as a participant in a clinical study, you can contact the patient rights ombudsman of your institution on this telephone number: LOCAL NUMBER RIGHTS OMBUDSMAN. If necessary, he/she can put you in contact with the ethics committee.

Title of the study:

A prospective, multicenter, randomized, parallel-group controlledtrial to compare conservative versus surgical treatment in peroneal nerve entrapment.

A comparative study for surgical and conservative treatment in foot drop due to peroneal nerve entrapment.

II Informed consent

Participant

I declare that I have been informed of the nature of the study, its purpose, its duration, any risks and benefits and what is expected of me. I have taken note of the information document and the appendices to this document.

I have had sufficient time to think about it and discuss it with a person of my choice, such as my GP or a member of my family.

I have had the opportunity to ask any questions that came to mind and have obtained a satisfactory response to my questions.

I understand that my participation in this study is voluntary and that I am free to end my participation in this study without this affecting my relationship with the therapeutic team in charge of my health.

I understand that data about me will be collected throughout my participation in this study and that the investigator and the sponsor of the study will guarantee the confidentiality of these data in accordance with applicable European and Belgian legislation. I understand that the performance of this study by UZ Leuven serves the general interest and that the processing of my personal data is necessary for the performance of this study.

I understand that my Belgian national number is collected and may be used by a trusted third party to link my study data to data from other sources (more information can be found in the appendices).

I agree to my GP or other specialists in charge of my health being informed of my participation in this clinical study.

I have received a copy of the information to the participant and the informed consent form.

Surname, first name and signature of the volunteer:

Date:

Investigator

I, the undersigned, investigator, confirm that I have verbally provided the necessary information about the study and have given the participant a copy of the information document.

I confirm that no pressure was applied to persuade the patient to agree to take part in the study and that I am willing to answer any additional questions if required.

I confirm that I operate in accordance with the ethical principles set out in the latest version of the “Helsinki Declaration”, the “Good Clinical Practices” and the Belgian Law of 7 May 2004 related to experiments on humans.

Surname, first name and signature of the investigator’s representative / investigator:<br><br><br><br>Date:

Title of the study:

A prospective, multicenter, randomized, parallel-group controlled trial to compare conservative versus surgical treatment in peroneal nerve entrapment.

A comparative study for surgical and conservative treatment in foot drop due to peroneal nerve entrapment.

III Supplementary information

1. Supplementary information on the timeline of the study: flow chart

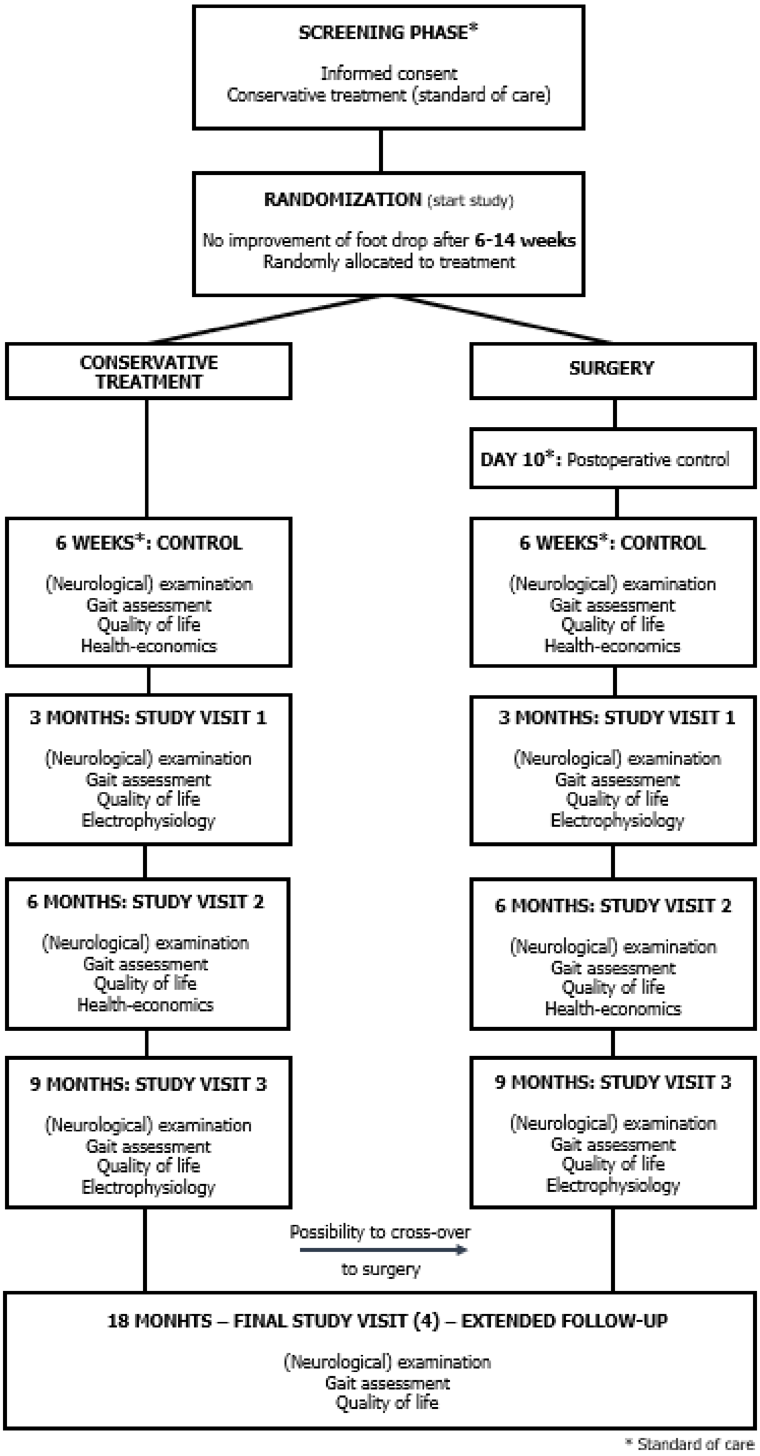

2: Supplementary information on the protection and the rights of the participant in a clinical study

Ethics Committee

This study has been reviewed by an independent Ethics Committee, namely the Ethics Committee of UZ Leuven, which has issued a favourable opinion [*after consulting with the Ethics Committees of each centre where this trial will be conducted, including NAME LOCAL ETHICS COMMITTEE*]. It is the task of the Ethics Committees to protect people who take part in a clinical trial. They make sure that your rights as a patient and as a participant in a clinical study are respected, that based on current knowledge, the balance between risks and benefits remains favourable to the participants, that the study is scientifically relevant and ethical.
You should not under any circumstances take the favourable opinion of the Ethics Committee as an incentive to take part in this study.

Voluntary participation

Before signing, do not hesitate to ask any questions you feel are appropriate. Take the time to discuss matters with a trusted person if you so wish.

Your participation in the study is voluntary and must remain free of any coercion: this means that you have the right not to take part in the study or to withdraw without giving a reason, even if you previously agreed to take part. Your decision will not affect your relationship with the investigator or the quality of your future therapeutic care.

However, it is advisable for your safety to inform the investigator if you have decided to stop taking part in the study.

If you agree to take part, you will sign the informed consent form. The investigator will also sign this form to confirm that he/she has provided you with the necessary information about the study. You will receive a copy of the form.

Costs associated with your participation

The trial is funded by the Belgian Healthcare Knowledge Centre (KCE). Hence, all expenses for study-related investigations are covered. Investigations and study visits that are considered standard of care, are not covered by study budget.

If you decide to take part in this study, this will not therefore involve any extra costs for you or your insurer. The visits and procedures identified as specific to the study in the description of the course of the study on page 3 to 4 or in the schedule on page 4 will be paid for by the sponsor. You may only be charged for the costs corresponding to the standard medical care in your clinical situation.

Guarantee of confidentiality

Your participation in the study means that you agree to the investigator collecting data about you and to the study sponsor using these data for research purposes and in connection with scientific and medical publications.

The processing of your personal data is necessary to achieve the scientific research purposes as set out herein.

Your data will be processed in accordance with the European General Data Protection Regulation (GDPR). UZ Leuven shall act as data controller for your data. You are entitled to ask the investigator what data are being collected about you and what is their use in connection with the study. This data concerns your current clinical situation but also some of your background, the results of examinations carried out within the context of care of your health in accordance with the current standards and

obviously the results of examinations required by the protocol. You have the right to inspect these data and correct them if they are incorrect[1].

The investigator has a duty of confidentiality vis-à-vis the data collected.

This means that he/she undertakes not only never to reveal your name in the context of a publication or conference but also that he/she will encode (your identity will be replaced by an ID code in the study) your data before sending them to the manager of the database of collected data (UZ LEUVEN).

The investigator and his/her team will therefore be the only ones to be able to establish a link between the data transmitted throughout the study and your medical records[2].

The personal data transmitted will not contain any combination of elements that might allow you to be identified[3].

For the study data manager designated by the sponsor, the data transmitted will not allow you to be identified. The latter is responsible for collecting the data gathered by all investigators taking part in the study, processing them and protecting them in accordance with the requirements of the Belgian law on the protection of privacy.

To verify the quality of the study, it is possible that your medical records will be examined by persons subject to professional secrecy and designated by the ethics committee, the sponsor of the study or an independent audit body. In any event, this examination of your medical records may only take place under the responsibility of the investigator and under the supervision of one of the collaborators designated by him/her.

The (encoded) study data will be able to be sent to Belgian or other regulatory authorities, to the relevant ethics committees, to other doctors and/or to organisations working in collaboration with the sponsor.

They will also be able to be sent to other sites of the sponsor in Belgium and in other countries where the standards in terms of the protection of personal data may be different or less stringent? As explained above, the transmitted data are encoded[4].

Your consent to take part in this study therefore also implies your consent to the use of your encoded medical data for the purposes described in this information form and to their transmission to the aforementioned people and authorities.

The sponsor undertakes only to use the data collected within the context of the study in which you are taking part.

If you withdraw your consent to take part in the study, to guarantee the validity of the research, the data encoded up to the point at which you withdraw will be retained. No new data may be sent to the sponsor.
If you have any questions relating to how your data are being processed, you may contact the investigator. The data protection officer in your hospital can be contacted as well: CONTACT INFORMATION LOCAL DPO.

Finally, if you have a complaint concerning the processing of your data, you can contact the Belgian supervisory authority who ensures that privacy is respected when personal data are processed.

The Belgian supervisory authority is called:
Data Protection Authority (DPA)
Drukpersstraat 35,
1000 Brussels
Tel. +32 2 274 48 00
e-mail: contact@apd-gba.be

---

[1] These rights are guaranteed by the European Data Protection Regulation (GDPR) and by the Law of 22 August 2002 on patient rights.

[2] For clinical trials, the law requires this link with your records to be retained for 20 years. In the case of a advanced therapy medicinal product using human biological material, this period will be a minimum of 30 years and a maximum of 50 years in accordance with the Belgian Law of 19 December 2008 on the use of human biological material and the applicable royal decrees.

[3] The database containing the results of the study will therefore not contain any combination of elements such as your initials, your gender and your full date of birth (dd/mm/yyyy).

[4] The sponsor then undertakes to respect the constraints of the European General Data Protection Regulation (GDPR) and the Belgian legislation on the protection of natural persons with regard to the processing of personal data.

Website: https://www.dataprotectionauthority.be

Where will your data be used?

The purpose of this study is to determine whether a particular treatment works as well or better than the current standard treatment in day-to-day practice. If the study shows that a treatment works as well or better, the study data will then be used to assess whether the cost of the treatment is proportionate to the expected health gain and improved quality of life.

Where will your data be used?

If you participate in this study, your data from this study may be used by the KCE which is funding the study or by similar public health research institutes in Europe for further analyses, e.g. to determine which of the studied treatments is preferable.

KCE is an independent public research centre that provides scientific advice on public health topics. The objective and tasks of the KCE are set out in Articles 262 to 268 of the Programme Law (I) of 24 December 2002. As part of these tasks, KCE must have access to certain personal data relating to the health of Belgian citizens and has the task of carrying out analyses based on coded data (pseudonymised data) in the public interest.

At the end of the study, the healthcare provider or healthcare institution of this study may be asked to transfer your national register number to the eHealth platform as a third party of trust, for pseudonomisation. This means that your national registry number will be replaced by a meaningless code. The eHealth-platform has the legal mandate to do this (Article 5 of the Act of 21 August 2008 establishing and organising the eHealth platform and various provisions).

Your identity will always be known only to your healthcare provider or healthcare institution. For pseudonymisation purposes, the eHealth platform temporarily has your national registration number at its disposal, but not your other data or the data from this study. KCE only has pseudonymised personal data without identity data.

In this way, for KCE, the data from this study can be linked to data from other sources, such as healthcare billing data, without KCE knowing your identity. But linking study data with other data for KCE can only be done under strict conditions and after a positive opinion from an independent organisation, the Information Security Committee (IVC). The IVC assesses whether the requested processing can be done correctly according to the applicable legislation. All decisions of the IVC are public and can be consulted on the website of the ICV (https://www.ehealth.fgov.be/ehealthplatform/nl/sectoraal-comite/documenten). KCE reports are also publicly available (https://kce.fgov.be/nl/publicaties/alle-rapporten) and contain only anonymous results.

Confidentiality of your data

Your data are processed in accordance with the Belgian law of 30 July 2018 on the protection of natural persons with regard to the processing of personal data and the Regulation (EU) 2016/679 of 27 April 2016, which entered into force on 25 May 2018, on the protection of natural persons with regard to the processing of personal data and on the free movement of such data.

All KCE researchers are legally bound by their professional duty of confidentiality.

If you have any questions or comments about the processing of your data, you can easily contact KCE as the data controller at info@kce.fgov.be or by letter to KCE, Avenue du Jardin Botanique 55, 1000 Brussels. If you wish, the KCE Data Protection Officer can provide you with more information on the protection of your personal data. You can contact him via e-mail (kce_dpo@kce.fgov.be).

If this should not prove sufficient, you may lodge a complaint about how your information is handled, with the Belgian supervisory authority responsible for enforcing data protection legislation: Data Protection Authority (GBA),Drukpersstraat 35, 1000 Brussels, or via e-mail (contact@apd-gba.be).

Insurance

Any participation in a clinical study involves a risk, however small it is. Even if there is no fault, the sponsor accepts responsibility for damage caused to the participant (or in the event of death, his/her dependants) and directly or indirectly linked to his/her participation in the study. The sponsor has taken out insurance for this responsibility[5].

You are therefore asked to report any new health problem to the investigator. He/she will be able to provide you with additional information concerning possible treatments.

[5] In accordance with Article 29 of the Belgian Law related to experiments on humans (7 May 2004)

If the investigator believes that a link with the study is possible (the insurance does not cover the natural progression of your disease or the known side effects of your normal treatment), he/she will inform the study sponsor, which will initiate the declaration procedure to the insurance company. The latter will appoint an expert - if it considers it necessary - to assess whether there is a link between your new health problems and the study.

In the event of disagreement either with the investigator or with the expert appointed by the insurance company and also whenever you feel it is appropriate, you or - in case of death - your dependants may bring proceedings against the insurer directly in Belgium.

The law provides that the insurer may be summoned to appear either before the judge of the location where the event giving rise to the damage occurred, or before the judge of your domicile, or before the judge of the insurer's registered offices.

Coordinates of the insurance:

Amlin Corporate Insurance

polisnr. 299.053.700

Tel.: Tel +32 (0)2 894 70 00

Coordinates of the broker:

Vanbreda Risk & Benefits

Plantin en Moretuslei 297, 2140 Antwerpen

Tel.: +32 3 217 55 74
